# Comparative Efficacy of Platelet-Rich Fibrin, Freeze-Dried Bone Allograft, or Spontaneous Healing for Alveolar Ridge Preservation: Systematic Review and Meta-Analysis

**DOI:** 10.3390/bioengineering12111253

**Published:** 2025-11-16

**Authors:** Abeer S. Al-Zawawi, Amani M. Basudan, Rand Osama Alkhani, Lamis Khalid Alraddadi, Shikha Fahad Bin-Muhayya, Layan Abdullah Alzuwayyid, Deemah Alsaeed, Eithar Ibrahim Alrosaa, Lana Mohammed Alrasheed, Muneerah Abduaziz Alfahad, Ghadeer Mohammed Almutairi, Jana Alawad, Wasan Saeed Koaban, Munirah Naeem Alsubaie, Sundar Ramalingam

**Affiliations:** 1 Department of Periodontics and Community Dentistry, College of Dentistry, King Saud University, Riyadh 11451, Saudi Arabia; aalzawawi@ksu.edu.sa (A.S.A.-Z.); abasudan@ksu.edu.sa (A.M.B.); 2 College of Dentistry, King Saud University, Riyadh 12372, Saudi Arabia; roalkhani@outlook.com (R.O.A.); lamees1423@hotmail.com (L.K.A.); shoshoalf65666@gmail.com (S.F.B.-M.); 3 College of Dentistry, Majmaah University, Al-Majmaah 15341, Saudi Arabia; layanabullah@gmail.com (L.A.A.); sdeemah23@gmail.com (D.A.); e.rawsa55@gmail.com (E.I.A.); lanamohammedd37@gmail.com (L.M.A.); meemaa1290@gmail.com (M.A.A.); ghaddrm44@gmail.com (G.M.A.); janaawad82@gmail.com (J.A.); 4 College of Dentistry, King Saud bin Abdulaziz University for Health Sciences, Riyadh 14611, Saudi Arabia; wasankoaban@gmail.com (W.S.K.); munera-als@hotmail.com (M.N.A.); 5 Department of Oral and Maxillofacial Surgery, College of Dentistry, King Saud University, Riyadh 11545, Saudi Arabia; 6 Maxillofacial Surgery Clinics, Dental University Hospital, King Saud University Medical City, Riyadh 12372, Saudi Arabia

**Keywords:** alveolar ridge preservation, platelet-rich fibrin, freeze-dried bone allograft, spontaneous healing, dental implants

## Abstract

Alveolar ridge preservation (ARP) is crucial for maintaining bone and soft-tissue integrity after tooth extraction, thereby facilitating future implant placement. Among various biomaterials, platelet-rich fibrin (PRF) and freeze-dried bone allograft (FDBA) are commonly used; however, their comparative effectiveness remains unclear. This systematic review and meta-analysis aimed to evaluate and compare the outcomes of PRF, FDBA, and spontaneous healing with blood clot in ARP, incorporating recent randomized controlled trials and comparative studies published up to June 2025. Electronic searches were conducted across multiple databases following the PRISMA 2020 guidelines, and the risk of bias was assessed using RoB-2 and ROBINS-I tools. Primary outcomes included changes in alveolar ridge height and width, while secondary outcomes encompassed histological, radiographic, implant-related, and patient-centered measures. Twenty studies were included for qualitative synthesis and sixteen for quantitative analysis. Meta-analyses showed no significant difference between PRF and FDBA in ridge height (SMD = −0.24; 95% CI: −0.56 to 0.08; *p* = 0.145) or width preservation (SMD = −0.16; 95% CI: −0.73 to 0.42; *p* = 0.597). PRF significantly reduced ridge height loss compared to spontaneous healing (SMD = −0.79; 95% CI: −1.33 to −0.25; *p* = 0.004) and enhanced histologic new bone formation (SMD = 1.43; 95% CI: 0.39 to 2.47; *p* = 0.007), while FDBA showed a non-significant trend toward benefit (SMD = −0.37; 95% CI: −0.86 to 0.11; *p* = 0.129). Moderate risk-of-bias and heterogeneity were observed among included studies. In conclusion, PRF and FDBA are both effective for alveolar ridge preservation, outperforming spontaneous healing. PRF offers biologically driven benefits in bone quality and soft-tissue healing, whereas FDBA provides greater structural stability. These findings suggest a promising clinical potential for PRF in improving bone quality at the implant site. Moreover, considering cost, preparation complexity, and site-specific needs, PRF may serve as a cost-effective, clinically favorable option for ARP. Future multi-center randomized trials with standardized PRF protocols and long-term follow-up are recommended.

## 1. Introduction

Alveolar ridge preservation (ARP) has become a cornerstone of modern implant dentistry, aiming to minimize post-extraction bone resorption and maintain the structural integrity of peri-implant tissues [1]. Clinical practice prior to the advent of ARP techniques involved instructing patients to follow post-surgical instructions that enabled the formation of a blood clot within the extraction socket [2]. This process of spontaneous healing progressed sequentially through the physiological steps of clot organization, granulation tissue formation, fibrous tissue ingrowth, osteoid deposition, and mineralization [3]. Nevertheless, the alveolar ridge undergoes significant dimensional changes due to physiological bone remodeling, which can compromise the success of subsequent implant placement [4]. To counteract these changes, various biomaterials have been employed, with freeze-dried bone allograft (FDBA) and platelet-rich fibrin (PRF) emerging as two of the most widely used options [5].

FDBA, a demineralized or mineralized bone graft, provides an osteoconductive scaffold that supports new bone formation [6]. PRF, an autologous platelet concentrate, enhances tissue regeneration through the sustained release of growth factors and cytokines [2,5,7]. Despite their widespread adoption, the comparative effectiveness of these materials in preserving peri-implant tissues remains a subject of ongoing debate [8,9]. The biological mechanisms underlying PRF and FDBA differ significantly, resulting in distinct clinical outcomes. While PRF leverages its bioactive components to accelerate soft tissue healing and modulate inflammation, FDBA relies on its structural properties to maintain ridge dimensions and promote osteogenesis [9,10,11]. Previous studies have reported conflicting results regarding their efficacy, with some suggesting superior bone preservation with FDBA and others highlighting the advantages of PRF in soft tissue regeneration [7]. These discrepancies may stem from variations in study design, patient populations, or outcome measures, underscoring the need for a systematic synthesis of the available evidence [1,2,5,7]. Moreover, the lack of consensus on optimal graft selection has left clinicians without clear guidance, which may affect treatment predictability and patient satisfaction.

A critical gap in the literature is the absence of a comprehensive meta-analysis comparing PRF and FDBA across multiple ARP outcomes pertinent to implant placement in the healed extraction socket, and how these techniques compare against the more conventional spontaneous healing with blood clot. While individual studies have examined specific aspects such as bone density or soft tissue thickness, no unified evaluation has integrated these dimensions to provide a holistic assessment [1,2,5,7,11,12,13,14]. Furthermore, existing reviews often focus on single biomaterials or fail to account for heterogeneity in clinical protocols, limiting their generalizability. Addressing these gaps is essential for refining clinical decision-making and optimizing ARP strategies.

Despite earlier reviews and meta-analyses comparing PRF, FDBA, and spontaneous healing for ARP, most of them are limited by narrow inclusion periods and excluded recent randomized controlled trials (RCTs) [2,5,15,16,17,18,19]. This review incorporates RCTs published up to mid-2025 to present the latest evidence. It endeavors to analyze histomorphometric and radiographic outcomes alongside patient-centered metrics for a comprehensive evaluation of alveolar ridge preservation. Additionally, by following PRISMA-2020 (Preferred Reporting Items for Systematic Reviews and Meta-Analyses) guidelines and using GRADE (Grading of Recommendations, Assessment, Development and Evaluations) for certainty assessment, this review offers a rigorous synthesis that enhances the understanding of PRF and FDBA efficacy for ARP.

The present review was based on the hypothesis that both PRF and FDBA would demonstrate superior ridge preservation compared to spontaneous healing, with PRF offering additional histologic and soft-tissue benefits. Accordingly, this review aims to evaluate the relative efficacy of PRF and FDBA for ARP and compare them to spontaneous socket healing, thereby informing evidence-based practice and guiding future research directions. By analyzing data from various clinical settings, this research aims to evaluate the effectiveness of PRF and FDBA in ARP for improving peri-implant outcomes. The findings of this review shall be of value to clinicians and researchers, providing a data-driven basis for graft selection in ARP.

## 2. Materials and Methods

### 2.1. Review Protocol

This systematic review and meta-analysis was designed according to the Preferred Reporting Items for Systematic Reviews and Meta-Analyses (PRISMA) 2020 guidelines to ensure methodological rigor and transparency [20]. The study protocol was registered in PROSPERO (ID: CRD42024571218). Being a systematic review and meta-analysis based exclusively on data extracted from previously published studies, ethical approval and informed consent requirements were waived. The following databases, namely, PubMed/MEDLINE, Cochrane Central Register of Controlled Trials (CENTRAL), Web of Science (All databases), and ScienceDirect (Elsevier), were primarily used for literature searching. Additionally, Google Scholar database was used as a supplementary source to identify non-indexed and gray literature. The literature search strategy along with respective search string for each database and the search results are shown in Supplemental Table S1.

Briefly, the search strings combined controlled vocabulary (e.g., MeSH terms) and free-text keywords, including but not limited to “platelet-rich fibrin”, “PRF”, “freeze-dried bone allograft”, “FDBA”, “spontaneous healing”, “blood clot”, “socket preservation”, “ridge preservation”, “alveolar ridge”, “extraction socket”, and “dental implants”. Studies obtained through literature search were exported to a reference management software (EndNote Version 15) for title and abstract screening, and removal of duplicates.

### 2.2. Research Question

The research question was structured based on the PICOS (Population, Intervention, Comparator, Outcomes, Study design) framework (Table 1).

Accordingly, the central research question of this review was, “In patients undergoing tooth extraction with socket preservation, how does the application of platelet-rich fibrin (PRF) compare to freeze-dried bone allograft (FDBA) and spontaneous healing with a blood clot in terms of effectiveness for alveolar ridge preservation?”

### 2.3. Inclusion and Exclusion Criteria

Studies were included in this review if they met the following criteria: (1) they were conducted on human subjects who had tooth extraction sites undergoing ridge or socket preservation, (2) they utilized isolated platelet-rich fibrin (PRF) as the intervention, (3) they compared the effects of PRF with either freeze-dried bone allograft (FDBA) or spontaneous healing (blood clot), (4) they reported at least one of the predefined outcomes of interest (clinical, radiographic, histological, or patient-reported outcomes), either qualitatively or quantitatively, (5) they were original research articles published in English between January 2010 and June 2025, and (6) they had a minimum follow-up period of 3 months.

Exclusion criteria primarily encompassed studies that combined PRF or FDBA with other biomaterials (e.g., xenografts, synthetic grafts, barrier membranes) without separate analysis of PRF/FDBA alone. Moreover, reviews, case reports, non-comparative studies, publications lacking primary data (e.g., editorials), and studies reporting translational animal models and in vitro experiments were excluded to maintain clinical relevance.

### 2.4. Study Selection Process and Data Extraction

Two calibrated independent reviewers (A.S.A. and A.M.B.), with a Cohen’s kappa score of 0.87, screened titles and abstracts, resolving discrepancies through discussion. The help of a third reviewer (S.R.) was sought to obtain consensus in case of unresolved discrepancies even after discussion. Full-text assessment followed the same protocol, with reasons for exclusion documented. Study quality and assessment for risk-of-bias were evaluated using the Cochrane Risk of Bias Tools, namely, RoB-2 for randomized trials and ROBINS-I for non-randomized studies of interventions [21,22]. In order to graphically represent the findings of the risk of bias assessment, the ROBVIS (Risk of Bias Visualization) tool was used [23].

The two reviewers independently extracted data using a standardized extraction form. The extracted variables included study objectives, demographic details (such as author, year, country, and design), sample size, both qualitative and quantitative details of the interventions and comparators, outcomes assessed (including radiographic, histological, clinical, and patient-reported outcomes), follow-up duration, and key findings. Given that the present study was a systematic review and meta-analysis, sample-size calculation was not necessary, and all studies that met the established inclusion criteria were analyzed.

### 2.5. Data Synthesis and Statistical Analysis

Extracted data were tabulated for qualitative synthesis and selected studies were subjected to quantitative analysis. A meta-analysis was conducted using Review Manager Software (RevMan) Version 5.4 (Copenhagen: The Nordic Cochrane Centre, The Cochrane Collaboration). This analysis included studies that provided data for specific outcomes, requiring a minimum of three studies for inclusion. The analysis included studies for both clinical and statistical heterogeneity. Clinical heterogeneity was assessed using a random-effects model to compare the outcomes observed post-treatment between the intervention and comparator groups, focusing on changes in alveolar bone dimensions, quantitative clinical results, radiographic and histological findings, as well as patient-centered outcomes. For the outcome effect measures, standardized mean differences (SMDs) with 95% confidence intervals (CIs) were calculated, and Forest plots were generated.

Since this meta-analysis was based on aggregated study-level data rather than individual measurements, normality testing was not applicable. Instead, for evaluating statistical heterogeneity, the chi-square test was applied, with a *p*-value of less than 0.05 considered statistically significant. Additionally, the I^2^ index was used further to characterize the degree of heterogeneity among the studies, wherein I^2^ values of 25%, 50% and 75% were considered low, moderate, and high heterogeneity, respectively. Publication bias during meta-analysis was evaluated using funnel plots and Egger’s regression test, when there were at least 10 studies per outcome. Wherever applicable, sensitivity analyses were performed to evaluate the robustness of the pooled estimates and to examine the impact of study design and potential sources of heterogeneity on the overall results.

In addition to quantitative synthesis, the Grading of Recommendations, Assessment, Development and Evaluations (GRADE) approach was applied to assess the overall certainty of evidence across key outcomes. The certainty of evidence was rated as high, moderate, low, or very low based on risk of bias, inconsistency, indirectness, imprecision, and publication bias [24]. A Summary of Findings (SoF) table was constructed to present pooled effect estimates, study counts, and certainty of evidence based on GRADE [24].

## 3. Results

### 3.1. Study Selection

**Figure 1:**
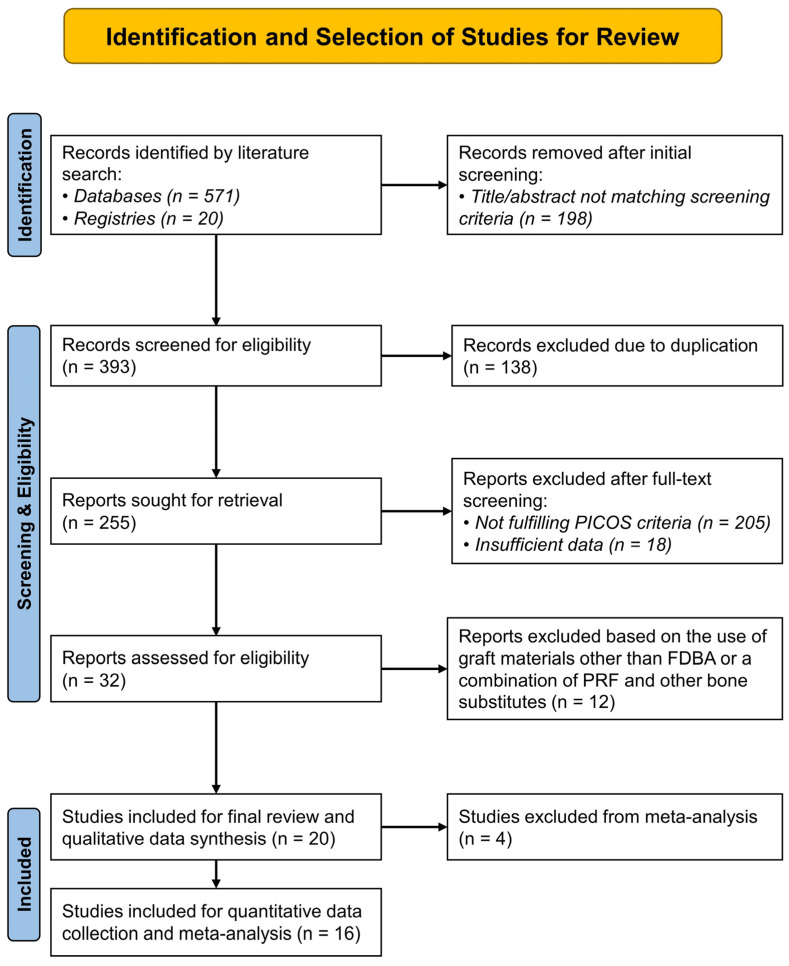
PRISMA (Preferred Reporting Items for Systematic Reviews and Meta-Analyses) flow diagram showing the sequence of identification and selection of studies for the systematic review. (PICOS—Population, Intervention, Comparator, Outcomes, Study design).

Altogether, 591 records were identified through literature searching, out of which 255 records were selected after initial screening and removal of duplicates. Finally, 20 studies were eligible and selected for qualitative synthesis. The primary reasons for excluding the remaining records were irrelevance to ARP and PICOS criteria (n = 205), use of graft materials other than FDBA or a combination of PRF and other bone substitutes (n = 12), and insufficient data (n = 18). Among the eligible studies, four were excluded from the quantitative synthesis and meta-analysis [9,25,26,27]. These included studies by Tajima et al. (2013) and Karagah et al. (2022), which reported data on bone dimensional changes after socket grafting with sinus elevation [26,27], and the study by Aldommari et al. (2025), which compared outcomes between PRF synthesized using different techniques [25]. In addition, the study by Azangookhiavi et al. (2024) was not considered for meta-analysis as it reported only on peri-implant hard and soft-tissue changes after ARP [9], and there was no other study reporting similar outcomes for comparison. Figure 1 shows the entire study selection process through a PRISMA flow diagram and Table 2 shows the qualitative data collected from the included studies.

### 3.2. Risk-of-Bias and Quality Assessment of the Included Studies

**Figure 2:**
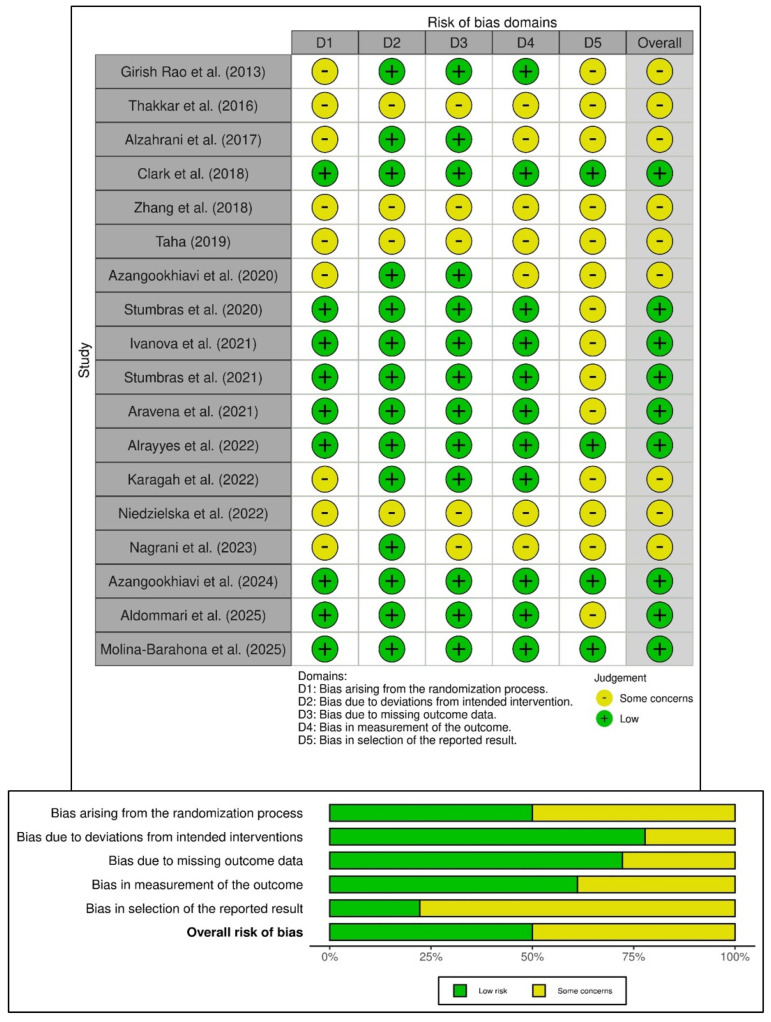
Risk of bias in the included randomized trials based on RoB-2 tool showing observed bias in each study judged across the five domains (**top**), and the summary of the risk-of-bias assessment (**bottom**) [3,9,10,25,26,28,29,30,31,32,33,34,35,36,37,38,39,41].

The risk of bias in the 18 included randomized controlled trials (RCTs) was assessed using the Cochrane Risk of Bias 2 (ROB-2) tool, summarized in Figure 2. Half of the RCTs showed a low risk of bias, while the remaining studies had some methodological concerns. Four RCTs (22.2%) showed low risk across all five domains [3,9,31,41], while four studies displayed concerns in all bias domains [29,32,33,38]. Specific biases included deviations from intended interventions (Domain 2—77.8%), missing outcome data (Domain 3—72.2%), and measurement bias (Domain 4—61.2%), indicating good adherence to interventions despite some follow-up issues. However, 50% of the RCTs showed bias from the randomization process (Domain 1) due to incomplete allocation concealment, and 77.8% had concerns regarding the selection of reported results (Domain 5) due to unclear blinding and selective reporting. Overall, half of the studies were low risk, but concerns in randomization and reporting warrant cautious interpretation. Notably, no studies were classified as high risk in any domain, thereby indicating moderate to high methodological quality in the evidence presented.

**Figure 3:**
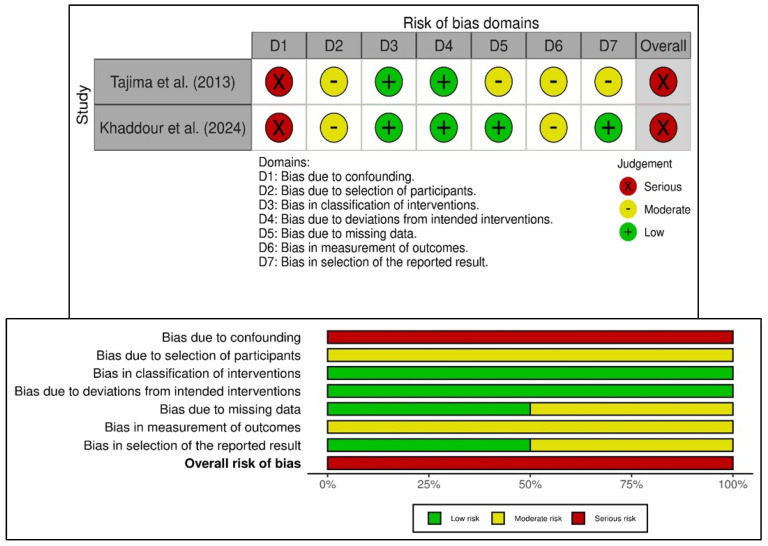
Risk of bias in the included non-randomized studies of interventions based on ROBINS-I tool showing observed bias in each study judged across the seven domains (**top**), and the summary of the risk-of-bias assessment (**bottom**) [27,40].

Additionally, this review assessed two non-randomized studies as having a serious risk of bias using the ROBINS-I tool (Figure 3) [27,40]. Both studies faced confounding due to non-randomized treatment allocation, which was influenced by patient or clinician preference. While the study by Tajima et al. (2013) [27] was limited by its retrospective design and incomplete baseline adjustment, the study reported by Khaddour et al. (2024) [40] had predefined outcomes but still experienced significant confounding and lacked blinding in outcome assessment. An overall serious risk of bias was identified in the non-randomized studies, not only because of confounding (Domain 1), but also due to biases in the selection of participants and the measurement of outcomes (Domains 2 and 6). Despite the low risk of bias related to the classification of interventions and deviations from planned interventions in both studies, the predominance of certain concerns in the bias domains highlights the methodological limitations of observational study designs. Although both studies offer valuable clinical insights into the use of platelet-rich fibrin for bone regeneration, their internal validity in the context of the overall evidence base is limited, warranting cautious interpretation of their conclusions.

### 3.3. Meta-Analysis—Reduction in Alveolar Ridge Height

**Figure 4:**
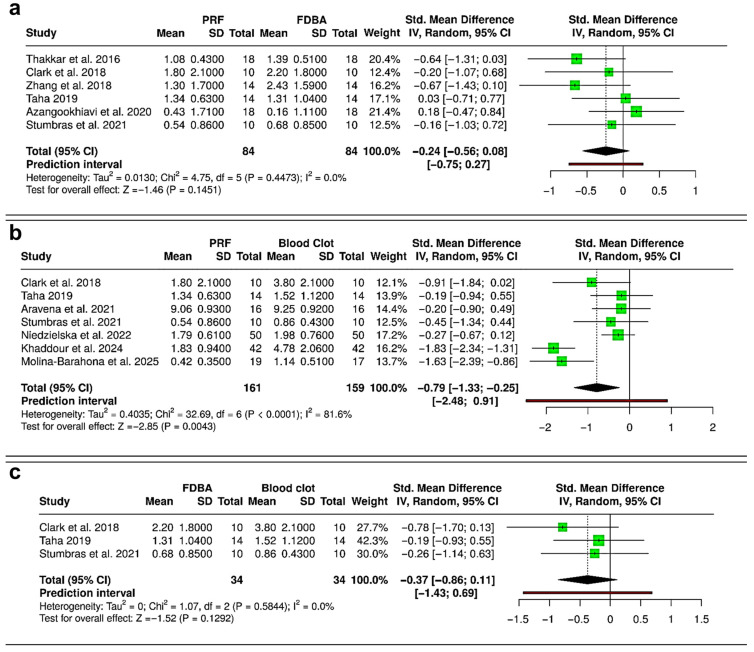
Forest plots showing the meta-analyses comparing different interventions for reduction in alveolar ridge bone height: (**a**) platelet-rich fibrin (PRF) versus freeze-dried bone allograft (FDBA) [10,29,31,32,33,36]; (**b**) PRF versus blood clot [31,33,36,37,38,40,41]; and (**c**) FDBA versus blood clot [31,33,36]. Data are presented as standardized mean differences (SMDs) with 95% confidence intervals (CIs) using a random-effects model. (SD—Standard deviation).

The meta-analysis comparing PRF and FDBA included six RCTs (168 extraction sockets—84 PRF, 84 FDBA) (Figure 4a). The pooled analysis demonstrated no statistically significant difference in ridge height reduction between PRF and FDBA (SMD = −0.24; 95% CI: −0.56 to 0.08; *p* = 0.1451). Heterogeneity was negligible (I^2^ = 0%), and the prediction interval (−0.75 to 0.27) suggests that future studies are unlikely to show consistent superiority of either material. When comparing PRF and spontaneous healing with blood clot (six RCTs and one cohort study; 320 extraction sockets—161 PRF, 159 blood clot), the results showed a significant reduction in ridge height loss favoring PRF (SMD = −0.79; 95% CI: −1.33 to −0.25; *p* = 0.0043), but there was considerable heterogeneity (I^2^ = 81.6%) (Figure 4b). The prediction interval (−2.48 to 0.91) suggested variable outcomes among individual studies, despite the overall trend favoring PRF. The comparison between FDBA and blood clot was based on three RCTs (68 extraction sockets—34 FDBA, 34 blood clot) (Figure 4c). The pooled effect suggested a non-significant trend toward reduced ridge height loss with FDBA (SMD = −0.37; 95% CI: −0.86 to 0.11; *p* = 0.1292). No evidence of heterogeneity was found (I^2^ = 0%). The prediction interval (−1.43 to 0.69) suggested that both favorable and null effects are possible in future studies. Overall, PRF and FDBA show similar results, with neither being superior. Both interventions reduce ridge height loss more effectively than spontaneous healing, with PRF having a significant advantage over blood clot, while FDBA shows a favorable but non-significant trend.

### 3.4. Meta-Analysis—Reduction in Alveolar Ridge Width

**Figure 5:**
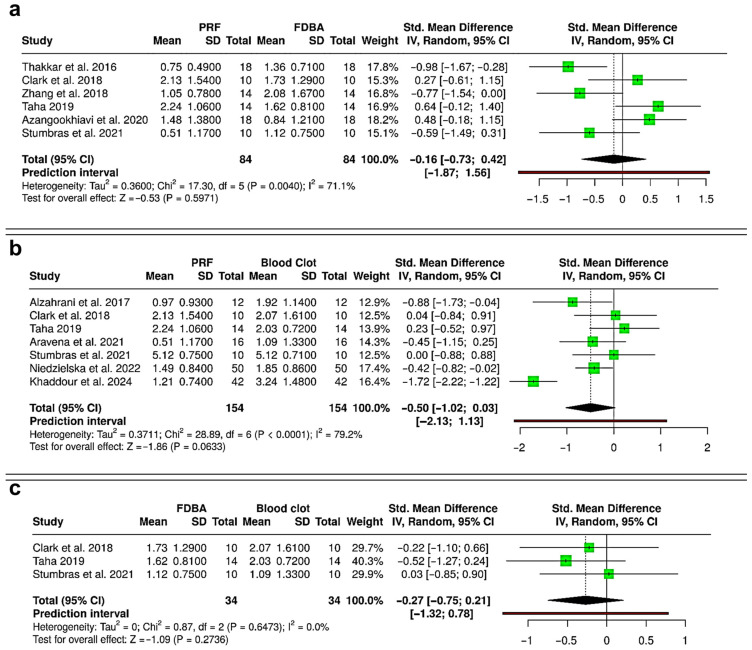
Forest plots showing the meta-analyses comparing different interventions for reduction in alveolar ridge bone width: (**a**) platelet-rich fibrin (PRF) versus freeze-dried bone allograft (FDBA) [10,29,31,32,33,36]; (**b**) PRF versus blood clot [30,31,33,36,37,38,40]; and (**c**) FDBA versus blood clot [31,33,36]. Data are presented as standardized mean differences (SMDs) with 95% confidence intervals (CIs) using a random-effects model. (SD—Standard deviation).

The meta-analysis comparing reduction in alveolar ridge width following interventions with PRF, FDBA, and blood clot is shown in Figure 5. Between PRF and FDBA (Six RCTs; 168 extraction sockets—84 PRF, 84 FDBA), the pooled results showed no statistically significant difference between the two interventions (SMD = −0.16; 95% CI: −0.73 to 0.42; *p* = 0.5971) (Figure 5a). Moderate heterogeneity was detected (I^2^ = 71.1%), and the prediction interval (−1.87 to 1.56) suggested wide variability, with possible outcomes favoring either PRF or FDBA in future studies. The pooled analysis comparing PRF versus blood clot (six RCTs and one cohort study; 308 extraction sockets—154 PRF, 154 blood clot) showed a non-significant trend favoring PRF for reducing ridge width loss (SMD = −0.50; 95% CI: −1.02 to 0.03; *p* = 0.0633), with substantial heterogeneity (I^2^ = 79.2%) (Figure 5b). The prediction interval (−2.13 to 1.13) highlighted that future studies may report either a benefit or a negligible effect for PRF compared with blood clot. Between FDBA and blood clot (three RCTs; 68 extraction sockets—34 FDBA, 34 blood clot), the pooled effect indicated no significant difference in ridge width reduction (SMD = −0.27; 95% CI: −0.75 to 0.21; *p* = 0.2736), with no observable heterogeneity (I^2^ = 0%) (Figure 5c). The prediction interval (−1.32 to 0.78) confirmed that both favorable and null outcomes remain possible. Overall, the evidence suggests that PRF and FDBA are similar in preserving ridge width, with neither showing superiority. Although both materials reduce ridge width loss more effectively than spontaneous healing, their results compared to blood clot healing were not statistically significant.

### 3.5. Meta-Analysis—Gain in Histometric Bone Area (%) in the Regenerated Bone

**Figure 6:**
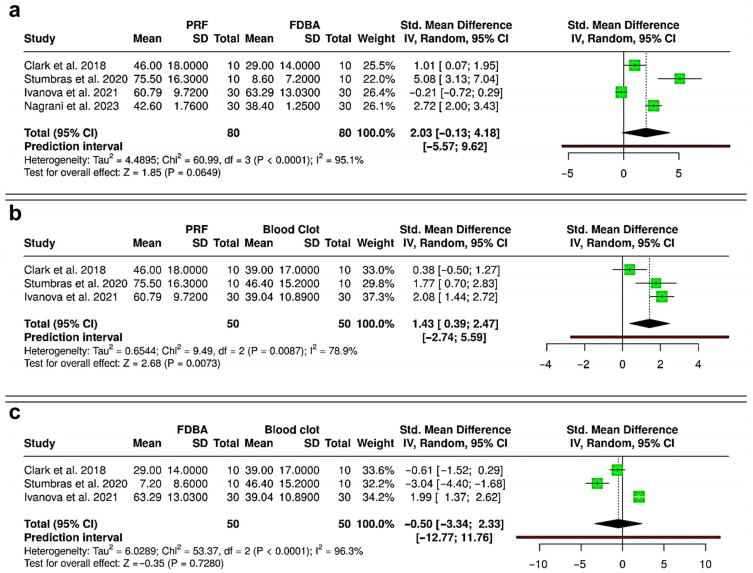
Forest plots showing the meta-analyses comparing different interventions for gain in histometric bone area (%): (**a**) platelet-rich fibrin (PRF) versus freeze-dried bone allograft (FDBA) [31,34,35,39]; (**b**) PRF versus blood clot [31,34,35]; and (**c**) FDBA versus blood clot [31,34,35]. Data are presented as standardized mean differences (SMDs) with 95% confidence intervals (CIs) using a random-effects model. (SD—Standard deviation).

Meta-analysis of gain in histological new bone area revealed variable outcomes across comparisons. Comparing PRF versus FDBA (four RCTs; 160 extraction sockets—80 PRF, 80 FDBA), the pooled analysis indicated a trend favoring PRF, with a standardized mean difference (SMD) of 2.03 (95% CI: −0.13 to 4.18; *p* = 0.065), but without statistical significance (Figure 6a). Heterogeneity was very high (I^2^ = 95.1%), and the prediction interval (−5.57 to 9.62) showed wide variability, suggesting that future studies could report outcomes in either direction. To compare both PRF and FDBA versus blood clot, three RCTs with 100 extraction sockets (50 PRF, 50 FDBA, and 50 blood clot) were analyzed. Between PRF and blood clot, the pooled effect showed a statistically significant benefit with PRF (SMD = 1.43; 95% CI: 0.39 to 2.47; *p* = 0.007) (Figure 6b). Heterogeneity was substantial (I^2^ = 78.9%), and prediction interval (−2.74 to 5.59) suggested that variability in effect sizes remains likely. Evaluating FDBA versus blood clot (Figure 6c), there was no significant difference between the interventions (SMD = −0.50; 95% CI: −3.34 to 2.33; *p* = 0.728), with extremely high heterogeneity (I^2^ = 96.3%), and the prediction interval (−12.77 to 11.76) reflected major inconsistency across studies. Overall, PRF showed a significant advantage over spontaneous healing with blood clot in enhancing histomorphometric bone area. Although PRF had a favorable trend compared to FDBA, it was not statistically significant due to high heterogeneity, while FDBA did not show a consistent benefit over blood clot.

### 3.6. Meta-Analysis—Gain in Radiographic Bone Fill (%) in the Regenerated Bone

**Figure 7:**
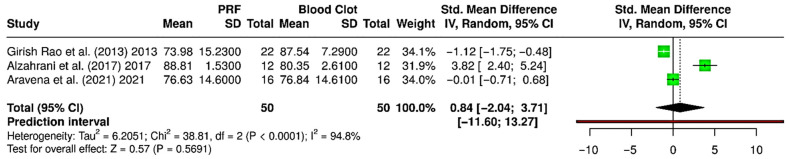
Forest plot showing the meta-analysis comparing platelet-rich fibrin (PRF) and blood clot for gain in radiographic bone fill (%) [28,30,37]. Data are presented as standardized mean differences (SMDs) with 95% confidence intervals (CIs) using a random-effects model. (SD—Standard deviation).

Quantitative data for radiographic bone fill was only available for PRF and blood clot interventions, so the meta-analysis compared only PRF to blood clot (three RCTs; 100 extraction sockets—50 PRF, 50 blood clot) [28,30,37] (Figure 7). The pooled analysis showed a standardized mean difference (SMD) of 0.84 [95% CI: −2.04 to 3.71; *p* = 0.569, indicating no significant difference in radiographic bone fill between PRF and blood clot. Extremely high heterogeneity was noted (I^2^ = 94.8%), suggesting variability among studies. The prediction interval was very wide, ranging from −11.60 to 13.27, indicating that future studies may yield varied results. These findings suggest that PRF does not consistently outperform spontaneous blood clot healing in promoting bone fill after socket preservation. While Alzahrani et al. (2017) showed a benefit of PRF [30], others found negligible or inferior outcomes compared to blood clot. This inconsistency indicates that the effectiveness of PRF may depend on case selection, surgical protocols, and patient variables.

### 3.7. Reduction in Soft-Tissue Socket Width

Although three studies reported on changes in soft-tissue socket width following ARP with either PRF, FDBA or blood clot healing [3,37,39], the comparisons were not subjected to meta-analysis, as only one study compared PRF vs. spontaneous healing [37] and only two studies compared PRF vs. FDBA [3,39]. Aravena et al. (2021) compared PRF (8.56 ± 0.96 mm) with spontaneous healing (7.94 ± 1.06 mm), showing a slightly greater reduction with PRF, though differences were marginal [37]. Alrayyes et al. (2022) demonstrated that PRF (7.43 ± 1.67 mm) resulted in greater socket width reduction compared to FDBA (5.77 ± 1.39 mm) [3]. Similarly, Nagrani et al. (2023) observed higher reduction with PRF (7.68 ± 0.84 mm) than with FDBA (7.03 ± 1.07 mm) [39]. Overall, FDBA appeared to better preserve socket width compared with PRF in two studies [3,39], while spontaneous healing yielded comparable outcomes to PRF in one study [37]. Due to fewer than three studies per direct comparison, meta-analysis was not feasible.

### 3.8. Sensitivity Analysis

**Figure 8:**
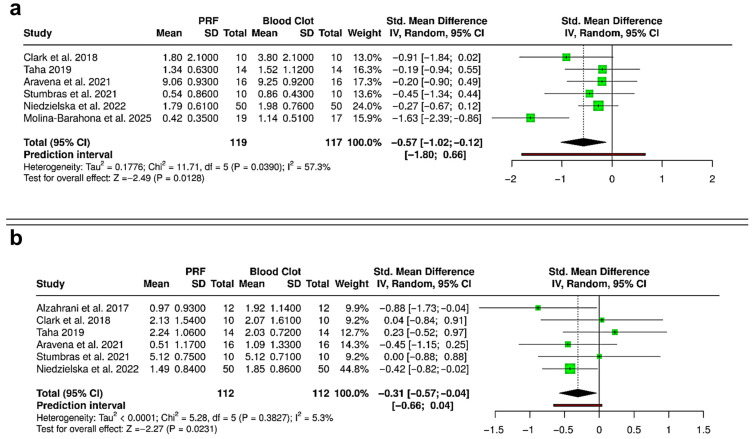
Forest plots showing the sensitivity analyses comparing platelet-rich fibrin (PRF) and blood clot for alveolar ridge preservation outcomes: (**a**) reduction in ridge height [31,33,36,37,38,41]; and (**b**) reduction in ridge width [30,31,33,36,37,38]. Analyses were performed using a random-effects model and presented as standardized mean differences (SMDs) with 95% confidence intervals (CIs), to compare results without inclusion of the non-randomized study by Khaddour et al. (2024) [40]. (SD—Standard deviation).

To evaluate the influence of the non-randomized study by Khaddour et al. (2024) on the pooled estimates, a sensitivity analysis was performed by excluding this study from the quantitative synthesis of ridge height and ridge width reduction (Figure 8) [40]. Inclusion of this study in the primary analysis showed that pooled standardized mean differences (SMDs) favored PRF over spontaneous healing for both parameters, but with high heterogeneity (I^2^ = 81.6% and 79.2%). After exclusion of the study, the overall direction of effect consistently favored PRF. However, the magnitude of the effect was attenuated, and between-study variability markedly decreased. For ridge height reduction (Figure 8a), the pooled SMD changed from −0.79 (95% CI: −1.33 to −0.25; *p* = 0.0043; I^2^ = 81.6%) to −0.57 (95% CI: −1.02 to −0.12; *p* = 0.0128; I^2^ = 57.3%). For ridge width reduction (Figure 8b), the pooled SMD changed from −0.50 (95% CI: −1.02 to 0.03; *p* = 0.0633; I^2^ = 79.2%) to −0.31 (95% CI: −0.57 to −0.04; *p* = 0.0231; I^2^ = 5.3%). Excluding the non-randomized study significantly reduced heterogeneity without changing the overall conclusions. The consistent effect direction indicates that including the non-randomized data did not bias the results, suggesting that the benefit of PRF in preserving alveolar ridge dimensions is strong across different study designs.

### 3.9. Summary of Findings and Certainty of Evidence

The Summary of Findings (SoF) table summarizes the pooled effect estimates, confidence intervals, and certainty of evidence for all primary and secondary outcomes, evaluated using the GRADE approach (Table 3). This offers an overview of the comparative effectiveness of PRF, FDBA, and spontaneous healing (blood clot) in alveolar ridge preservation. Moderate-certainty evidence indicated that PRF was associated with a meaningful reduction in ridge height loss and a consistent improvement in histometric new bone formation compared with spontaneous healing, reflecting both biological and clinical advantages. In contrast, comparisons involving FDBA were supported by a low to very low certainty of evidence, showing small and statistically non-significant effects on ridge dimensions and inconsistent results for new bone formation. Radiographic outcomes demonstrated wide variability and very low certainty, limiting their interpretability. Collectively, these findings suggest a more consistent and clinically relevant benefit for PRF, whereas evidence for FDBA remains inconclusive due to heterogeneity, imprecision, and methodological limitations among the included studies.

### 3.10. Differences in PRF Preparation Protocols Among Included Studies

The evaluation of PRF preparation protocols showed significant heterogeneity due to differences in techniques, additives, centrifugation parameters (speed, time, G-force), and product morphology (Table 4). Most PRF protocols, including leukocyte- and platelet-rich fibrin (L-PRF), utilize the patient’s whole blood in sterile, additive-free tubes, relying on the natural coagulation process during centrifugation. This absence of anticoagulants is a hallmark of second-generation platelet concentrates [11]. However, Girish Rao et al. incorporated an anticoagulant (acidulated citrate dextrose, ACD), necessitating the subsequent addition of Calcium Gluconate to promote coagulation and gel formation, distinguishing it from typical PRF methods [28]. Rotational speed (RPM) and preparation duration are crucial for determining the final product and vary significantly across studies. Most protocols followed the original Choukroun technique, operating at 2700 to 3000 rpm for about 10 to 12 min [13]. The high G-force centrifugation technique concentrates platelets and leukocytes into a dense L-PRF matrix, while A-PRF (Advanced PRF) protocols used a “low-speed concept” to enhance growth factor distribution, typically at 1300 rpm for 8 min [31], or for 14 min [3,40]. Similarly, the technique for injectable PRF synthesis used lower speed and shorter time, around 700 rpm for three minutes, producing a liquid top layer suitable for mixing with bone graft (sticky bone) [39]. Alternatively, specialized PRF concentrates, like Plasma Rich in Growth Factor (PRGF), used specific tubes and centrifugation protocols (e.g., PRGF System Centrífuge-IV) to separate plasma into distinct fractions (F1 and F2), differing from other PRF methods [34,36].

In addition to the differences mentioned, there were inconsistencies in blood volume, tube types, and products. Most preparations used sterile glass tubes, but Titanium-Prepared PRF (T-PRF) utilized specialized Grade IV titanium tubes. This material difference was thought to enhance platelet activation, producing a denser fibrin network and extended growth factor release compared to standard L-PRF prepared in glass tubes [25]. Similarly, the resultant PRF varied in form from a hard, elastic membrane and a re-coagulated gel to a dense clot and a liquid/semi-liquid product for making sticky bone with FDBA.

## 4. Discussion

### 4.1. Outcomes-Based Summary and Inference

The synthesis of evidence from the 20 selected studies reveals distinct patterns in the comparative effectiveness of PRF, FDBA, and spontaneous blood clot healing for peri-implant tissue preservation. Results of the meta-analysis demonstrate that both PRF and FDBA show a comparable performance in terms of preserving ridge height and width following extraction, with no significant differences observed between the two interventions. This suggests that the biological benefits of PRF and the space-maintaining properties of FDBA may result in similar clinical outcomes in terms of dimensional stability [10,15,31,42]. Importantly, PRF was found to have a significant effect in reducing ridge height loss compared with spontaneous healing, whereas FDBA showed only a non-significant trend in the same direction (Figure 4). In contrast, except for a non-significant trend favoring PRF over blood clot, no statistically significant differences were observable in terms of alveolar ridge width loss while comparing PRF, FDBA and spontaneous healing (Figure 5). These findings indicate that both interventions may be advantageous over blood clot healing, with PRF exhibiting more substantial evidence for ridge height and width preservation [17,42,43].

Histological outcomes identified through the present review further highlight the potential benefits of PRF [18]. The pooled analysis revealed a significant gain in new bone area when PRF was compared with spontaneous healing, underscoring its role in enhancing osteogenesis through the release of autologous growth factors [5,18,44,45,46]. While PRF also showed a favorable trend over FDBA in histometric bone formation, the result did not reach statistical significance due to high heterogeneity (Figure 6). Interestingly, FDBA did not demonstrate any consistent histological advantage over spontaneous healing, raising questions about its contribution to in situ bone regeneration versus space maintenance [35,39,45]. On the other hand, radiographic outcomes were less conclusive (Figure 7). No significant difference was found between PRF and spontaneous healing in terms of radiographic bone fill, with substantial heterogeneity among the included studies [28,30,37]. This inconsistency suggests that radiographic measures may be less sensitive to early or subtle biological effects, or that methodological variability across trials could influence outcomes [28,29,30,44,47,48].

Regarding changes in soft-tissue socket dimensions, the available evidence suggests differential outcomes depending on the intervention used. Alrayyes et al. (2022) and Nagrani et al. (2023) reported that FDBA is more effective than PRF in limiting socket width reduction, consistent with the osteoconductive and space-maintaining properties of bone grafts [3,39]. In contrast, Aravena et al. (2021) observed reductions in soft-tissue socket width that were similar between PRF and natural healing [37]. This finding implies that PRF’s impact on the stability of soft-tissue dimensions might be limited [13,49]. However, the small number of studies, with fewer than three available for each direct comparison, and heterogeneity in study design preclude firm conclusions, underscoring the need for further studies comparing PRF, FDBA, and spontaneous healing for soft-tissue outcomes in ARP [14,50].

### 4.2. Clinical Significance

The findings from this meta-analysis and systematic review are substantial, as both PRF and FDBA are effective strategies for preserving alveolar ridge height following tooth extraction, with comparable outcomes [51]. While PRF did not outperform FDBA, it demonstrated clear superiority over spontaneous healing with a blood clot, emphasizing its regenerative potential and ability to mitigate post-extraction ridge resorption [2,7,15,19,51,52]. For clinicians focusing on soft-tissue recovery and wound healing, particularly during implant placement in the esthetic zone, PRF is beneficial in reducing postoperative morbidity and enhancing mucosal integration [13,16,25,49,50,53]. However, it may not be effective for maintaining ridge dimensions [14]. In contrast, FDBA could be preferable in scenarios requiring volumetric stability, such as posterior ridge preservation or large extraction defects [14,40,41]. Theoretically, these findings enhance the conceptual framework for achieving success in ARP, which includes not just the quantity of bone but also its quality and the integration of soft tissue [1,12,14,32,53]. From a clinical decision-making perspective, PRF may be preferred in cases prioritizing biologically driven regeneration, soft-tissue healing, and cost-effectiveness, especially in anterior regions or when autologous materials are desired [49]. Conversely, FDBA may be selected when greater dimensional stability and mechanical support are required, such as in posterior regions or large extraction defects [54]. Selection should thus be based on patient-specific needs, surgical objectives, and material availability [1,54].

The advantage of PRF lies in its autologous, cost-effective, and minimally invasive preparation, making it an attractive alternative when bone substitutes are unavailable or contraindicated [5,9,44,55]. Conversely, FDBA remains a well-established option, particularly when a scaffold is required for defect stability [42,47]. The non-significant but favorable trend of FDBA over blood clot supports its continued clinical use [34,36]. In clinical decision-making, both PRF and FDBA are reliable options for ridge height preservation, with PRF offering the dual benefits of regeneration and patient acceptance due to its autologous nature [5,44]. At the same time, FDBA provides structural stability and proven long-term outcomes [37,40]. Clinically, these findings suggest that both PRF and FDBA are reliable materials for socket preservation, as they limit ridge width reduction [52,54]. The absence of significant differences between them suggests that clinicians may select either material based on availability, patient preference, cost, and biological considerations [14,15,49]. Despite a lack of statistical significance in comparisons between PRF and FDBA, which underscores the variability in clinical outcomes, PRF is preferred due to its low cost, minimal invasiveness, and ability to encourage natural regeneration [15,47]. However, FDBA may be chosen for its reliability as a scaffold to support bone regeneration [8,31]. Either way, it is important to note that depending solely on blood clot healing carries a higher risk of ridge width loss, which could complicate future implant placements [30,31,37].

From a perspective of vital bone formation in the ARP site, the significant benefit of PRF over a blood clot lies in its high concentration of growth factors and biologic mediators [31,35,45]. Furthermore, the lack of consistent superiority of FDBA over blood clot raises questions regarding its histological advantage [31,34,36]. While FDBA provides scaffold stability and is beneficial in maintaining alveolar contour, its contribution to vital bone formation appears less predictable than PRF [14,54]. Clinicians may therefore consider PRF as a reliable alternative or adjunct to grafting, especially in cases where cost, availability, or patient preference favors the use of autologous materials [13,14]. However, the variability in outcomes across studies highlights the need for further well-controlled trials.

### 4.3. Certainty of Reported Evidence

The GRADE assessment of the present meta-analysis underscores the current limitations of the evidence base. While moderate-certainty evidence supports the role of PRF in reducing ridge height loss and improving bone quality compared with spontaneous healing, the certainty of evidence for FDBA remains low due to heterogeneity across studies, methodological shortcomings, and small sample sizes (Table 3). The very low certainty associated with radiographic bone fill outcomes reflects considerable inconsistency, making it difficult to draw definitive conclusions [28,30,37]. These findings emphasize the importance of designing multi-center, long-term RCTs with standardized PRF preparation protocols and consistent outcome reporting [49], as this would strengthen the certainty of evidence and facilitate reliable clinical recommendations.

The current heterogeneity in centrifugation methods, follow-up durations, and outcome assessments undermines the comparability of results across studies [56]. Harmonizing PRF preparation techniques and applying consistent measurement protocols will reduce bias, allowing for a more straightforward interpretation of its regenerative potential [5,56]. Multi-center trials with follow-up periods extending beyond five years are crucial for capturing the temporal dynamics of graft resorption, soft-tissue adaptation, and implant stability [5,11,56]. Such studies would not only refine clinical guidelines for alveolar ridge preservation but also facilitate the development of patient-centered, evidence-based algorithms for biomaterial selection [54].

### 4.4. Study Limitations

Several methodological limitations temper the generalizability of the findings of the present review and meta-analysis. Primarily, there is high heterogeneity across studies, which confounds direct comparisons. For instance, differences in PRF preparation methods (e.g., centrifugation speed, fibrin concentration) may influence its biological potency and its osteoinductive potential [5,56]. Similarly, the predominance of short-term follow-ups (3–12 months) limits insights into the assessment of regenerated tissues, long-term tissue stability, FDBA’s resorption kinetics, and PRF’s sustained bioactive effects [8,57]. Although imperative, subgroup or meta-regression analyses were not feasible due to significant variability in PRF preparation methods, centrifugation protocols, and follow-up durations across studies (Table 2 and Table 4). There were also issues about the risk of bias in the included studies. Approximately half of the included randomized trials raised concerns related to randomization and selective reporting (Figure 2). The two non-randomized studies demonstrated a serious overall risk of bias due to confounding and limitations in outcome measurement (Figure 3). These methodological shortcomings necessitate a cautious interpretation of the synthesized outcomes, which should be considered when interpreting pooled results.

Another critical factor influencing clinical outcomes is the heterogeneity of patient populations (Table 2). Smoking status, systemic conditions such as diabetes, and periodontal phenotype may modulate the regenerative response differently with PRF and FDBA [3,7]. For example, studies in smokers suggested PRF’s bioactive matrix enhanced soft tissue closure and reduced complications, whereas FDBA’s benefits were less pronounced under compromised healing conditions [3,58]. Such findings point toward a future of patient-tailored ARP strategies rather than a one-size-fits-all approach [54]. For example, diabetic patients might benefit more from PRF’s anti-inflammatory properties, whereas those with compromised healing could require FDBA’s predictable volumetric maintenance [53]. Furthermore, exclusion of non-English or gray literature may have introduced selection bias.

Lastly, due to paucity of quantitative data, a non-randomized study with high risk of bias was included in the meta-analysis [40]. A sensitivity analysis excluding this study showed consistent results with reduced heterogeneity, confirming the advantage of PRF in minimizing reduction in alveolar ridge height and width, unaffected by study design.

### 4.5. Future Directions

Future studies should address three critical gaps identified in this review. First, randomized controlled trials with extended follow-ups (more than 5 years) are needed to evaluate the temporal dynamics of bone remodeling and soft tissue adaptation [8,57]. Second, mechanistic studies could elucidate how PRF’s growth factor release profiles (e.g., TGF-β1, IGF-1) interact with FDBA’s mineralized matrix to influence osteogenesis, a synergy that remains underexplored [35]. Third, cost-effectiveness analyses would help contextualize clinical decision-making, as PRF’s autologous nature may reduce material costs but increase procedural complexity compared to FDBA [25]. Emerging technologies such as 3D bioprinting or gene-activated matrices could further refine these materials by combining FDBA’s structural integrity with PRF’s biological activity [5,39,42].

Importantly, beyond biological and dimensional outcomes, patient-centered measures such as postoperative pain, swelling, and satisfaction should be incorporated in future trials [3,44,56]. PRF, being autologous, may enhance patient acceptance and reduce material-related concerns, whereas FDBA offers predictable scaffold stability but may be limited by cost or patient reluctance toward allogenic materials [13,14,56]. Integrating these factors into outcome assessments would align ARP research more closely with real-world clinical decision-making [59].

## 5. Conclusions

This systematic review and meta-analysis provides a comprehensive comparison of PRF, FDBA, and spontaneous healing in alveolar ridge preservation. The evidence indicates that PRF and FDBA perform similarly in preserving ridge dimensions, with neither material showing consistent superiority across all outcomes. PRF demonstrated significant benefits over spontaneous healing in reducing ridge height loss and enhancing histological bone formation, highlighting its biological potential to support regeneration. FDBA, in contrast, showed a tendency to maintain socket width better, reflecting its structural role as a space-maintaining graft. However, radiographic outcomes were inconclusive, and high heterogeneity across several analyses limits the strength of these conclusions.

From a clinical and translational perspective, these findings suggest that material selection should be individualized, with PRF favored where biologically driven regeneration is prioritized, and FDBA considered when dimensional stability is critical. PRF serves as a cost-effective, biologically favorable option emphasizing soft-tissue regeneration, whereas FDBA provides superior structural stability for maintaining ridge dimensions. Future multi-center randomized trials employing standardized PRF preparation protocols, long-term follow-up, and cost-effectiveness analyses are essential to guide evidence-based clinical decision-making.

## Figures and Tables

**Table 1:** PICOS (Population, Intervention, Comparator, Outcomes, Study design) criteria for framing the research question.

PICOS Criterion	Description
Population (P)	Human patients undergoing tooth extraction and subsequent alveolar ridge/socket preservation prior to implant placement
Intervention (I)	Socket preservation with platelet-rich fibrin (PRF)
Comparator (C)	Socket preservation with freeze-dried bone allograft (FDBA) and/or spontaneous healing with blood clot only
Outcomes (O)	Primary quantitative outcome measures: Dimensional change in alveolar bone height and width Secondary outcome measures: Quantitative histological and radiological evaluation for bone formation, reduction in soft-tissue socket width, post-implant stability, marginal bone loss, and patient-centered outcomes
Study design (S)	Randomized controlled trials (RCTs), comparative cohort studies and controlled clinical trials (CCTs)

**Table 2:** Qualitative Data Synthesis from the Included Studies.

Author (Year)	Study Design	Study Objective	Intervention vs. Comparator	Sample Size Per Group (Sockets)	Follow-Up Period (in Months)	Key Outcome
Tajima et al. (2013) [27] *	Prospective Interventional study	Assessed socket grafting and sinus floor augmentation along with simultaneous implant placement using PRF as the sole grafting material.	PRF vs. none	17	6	Sinus elevation with PRF supported natural bone regeneration during simultaneous implant placement.
Girish Rao et al. (2013) [28]	RCT	Examined the effect of autologous PRF on bone regeneration after tooth extraction.	PRF vs. spontaneous healing	22	6	PRF enhanced postoperative bone regeneration and density, supporting its role in hard tissue healing.
Thakkar et al. (2016) [29]	RCT	Evaluated bone fill in extraction sockets using FDBA alone and PRF, based on clinical and radiographic outcomes.	PRF vs. FDBA	18	6	PRF achieved comparable socket preservation outcomes to FDBA and may serve as an adjunctive material.
Alzahrani et al. (2017) [30]	RCT	Assessed socket healing clinically and radiographically when treated with autologous PRF.	PRF vs. spontaneous healing	12	3	PRF yielded greater ridge width maintenance and radiographic bone fill than spontaneous healing.
Clark et al. (2018) [31]	RCT	Compared PRF and FDBA for their effectiveness in vital bone formation and maintenance of alveolar ridge dimensions.	PRF vs. FDBA vs. spontaneous healing	10	4	Both PRF alone and PRF with FDBA were effective for alveolar ridge preservation.
Zhang et al. (2018) [32]	RCT	Examined the clinical efficacy of PRF in preserving alveolar ridge architecture after tooth extraction.	PRF vs. FDBA	14	3	PRF enhanced bone quality and accelerated bone formation, though it did not significantly reduce socket resorption.
Taha (2019) [33]	RCT	Investigated dimensional changes in the esthetic zone following tooth extraction when PRF was used as the only grafting material.	PRF vs. FDBA vs. spontaneous healing	14	6	Greater bone loss was observed with PRF compared to FDBA; PRF showed no short-term additive effect in limiting resorption.
Azangookhiavi et al. (2020) [10]	RCT	Compared clinical outcomes of alveolar ridge preservation using FDBA versus PRF after tooth extraction.	PRF vs. FDBA	18	3	PRF demonstrated acceptable, cost-effective efficacy for ridge preservation with results comparable to FDBA.
Stumbras et al. (2020) [34]	RCT	Compared histomorphometric outcomes of bone regeneration using PRF, bone substitutes, and spontaneous healing.	PRF vs. FDBA vs. spontaneous healing	10	3	PRF provided the most favorable histological outcomes for bone regeneration in the esthetic zone.
Ivanova et al. (2021) [35]	RCT	Evaluated histomorphometric results of socket preservation using FDBA versus PRF.	PRF vs. FDBA vs. spontaneous healing	30	4	Both PRF and FDBA promoted significantly greater vital bone formation than spontaneous healing.
Stumbras et al. (2021) [36]	RCT	Compared three-dimensional bone changes in ridge preservation with PRF, bone substitutes, and spontaneous healing.	PRF vs. FDBA vs. spontaneous healing	10	3	PRF and FDBA both reduced horizontal and vertical bone changes after extraction.
Aravena et al. (2021) [37]	RCT	Compared PRF filling with natural blood clot healing for alveolar ridge preservation.	PRF vs. spontaneous healing	16	3	PRF and spontaneous healing showed similar outcomes for wound healing and bone formation dimensions.
Alrayyes et al. (2022) [3]	RCT	Assessed the effect of PRF on socket wound healing in smokers.	PRF vs. FDBA	10	3	In smokers, PRF improved wound closure, healing, and reduced postoperative adverse effects.
Karagah et al. (2022) [26] *	RCT	Compared socket grafting and sinus floor augmentation using PRF versus FDBA with respect to stability of one-stage dental implants.	PRF vs. FDBA	10	6	PRF outperformed FDBA in maintaining implant stability after sinus lift and grafting.
Niedzielska et al. (2022) [38]	RCT	Investigated the role of PRF in preventing alveolar ridge height and width reduction after extraction.	PRF vs. spontaneous healing	50	6	PRF improved soft tissue healing, bone density, and reduced alveolar process atrophy at extraction sites.
Nagrani et al. (2023) [39]	RCT	Investigated the combined use of injectable PRF and bone graft for socket preservation.	PRF vs. FDBA	30	9	Injectable PRF aided socket healing, preserved crestal bone levels, and supported implant placement.
Azangookhiavi et al. (2024) [9] *	RCT	Evaluated peri-implant soft and hard tissue changes following ridge preservation with PRF versus FDBA.	PRF vs. FDBA	20	12 (post-implant loading)	PRF achieved similar peri-implant outcomes to FDBA, except for greater gingival recession after one year.
Khaddour et al. (2024) [40]	Retrospective cohort study	Compared alveolar dimensional changes after extraction between PRF and spontaneous healing using pre-implant planning data.	PRF vs. spontaneous healing	42	3	PRF reduced post-extraction bone volume loss compared with spontaneous healing.
Aldommari et al. (2025) [25] *	RCT	Compared titanium-prepared PRF (T-PRF), leukocyte-rich PRF (L-PRF), and spontaneous healing for alveolar ridge preservation.	PRF vs. spontaneous healing	10	4	PRF preserved ridge dimensions, increased bone density, and improved soft-tissue healing versus spontaneous healing.
Molina-Barahona et al. (2025) [41]	RCT	Evaluated the effect of PRF on maintaining alveolar bone dimensions post-extraction using CBCT.	PRF vs. spontaneous healing	19 (PRF)/17 (Blood clot)	4	PRF enhanced short-term alveolar height preservation, bone density, and ridge maintenance, though not all differences were statistically significant.

* Studies excluded from quantitative data collection and meta-analysis. RCT—Randomized controlled trial; PRF—Platelet-rich fibrin: FDBA—Freeze-dried bone allograft; CBCT—Cone beam computed tomography; ARP—Alveolar ridge preservation; T-PRF—Titanium-prepared platelet-rich fibrin; L-PRF—Leukocyte and platelet-rich fibrin.

**Table 3:** Summary of findings (SoF) table presenting the comparative effectiveness of platelet-rich fibrin (PRF), freeze-dried bone allograft (FDBA), and spontaneous healing (blood clot) in alveolar ridge preservation. Relative effects are expressed as standardized mean differences (SMDs) with 95% confidence intervals (CIs), and the certainty of evidence was rated using the GRADE (Grading of Recommendations, Assessment, Development and Evaluations) approach.

Outcome	Intervention vs. Comparator (n)	Relative Effect (SMD, 95% CI)	Certainty of Evidence	Comments
Reduction in ridge height	PRF vs. FDBA (168 sockets)	−0.24 (−0.56 to 0.08)	Moderate	No difference; both interventions effective.
	PRF vs. Blood clot (320 sockets)	−0.79 (−1.33 to −0.25)	Moderate	Significant benefit for PRF; high heterogeneity (I^2^ = 81.6%).
	FDBA vs. Blood clot (68 sockets)	−0.37 (−0.86 to 0.11)	Low	Trend favoring FDBA; not significant.
Reduction in ridge width	PRF vs. FDBA (168 sockets)	−0.16 (−0.73 to 0.42)	Low	No difference; moderate heterogeneity.
	PRF vs. Blood clot (308 sockets)	−0.50 (−1.02 to 0.03)	Low	Trend favoring PRF; not significant.
	FDBA vs. Blood clot (68 sockets)	−0.27 (−0.75 to 0.21)	Low	No significant benefit.
Histometric new bone area (%)	PRF vs. FDBA (160 sockets)	2.03 (−0.13 to 4.18)	Low	Trend favoring PRF; very high heterogeneity (I^2^ = 95%).
	PRF vs. Blood clot (100 sockets)	1.43 (0.39 to 2.47)	Moderate	PRF significantly improved new bone formation.
	FDBA vs. Blood clot (100 sockets)	−0.50 (−3.34 to 2.33)	Very low	No consistent benefit of FDBA.
Radiographic bone fill (%)	PRF vs. Blood clot (100 sockets)	0.84 (−2.04 to 3.71)	Very low	No consistent benefit; wide prediction interval.

SMD—Standardized mean difference; CI—Confidence interval; PRF—Platelet-rich fibrin; FDBA—Freeze-dried bone allograft; RCT—Randomized controlled trial.

**Table 4:** Summary of platelet-rich fibrin preparation protocols in the included studies. Detailed procedural parameters for each individual study are provided in Supplementary Table S1.

Author (Year)	PRF Type	Blood Volume (mL)	Centrifugation Parameters	Key Characteristics/Technique Summary
Girish Rao et al. (2013) [28]	Standard PRF (Clot/Gel/Membrane)	5 (including 0.5 mL ACD)	20 min at 360–400 rpm	Conventional PRF clots/membranes prepared by immediate centrifugation; fibrin clot separated and used as membrane or graft.
Tajima et al. (2013) [27] Thakkar et al. (2016) [29] Alzahrani et al. (2017) [30] Zhang et al. (2018) [32] Taha (2019) [33] Azangookhiavi et al. (2020, 2024) [9,10] Ivanova et al. (2021) [35] Niedzielska et al. (2022) [38] Molina-Barahona et al. (2025) [41]		9–20	10–12 min at ~2700–3000 rpm (≈400–700× *g*)	
Clark et al. (2018) [31] Alrayyes et al. (2022) [3] Khaddour et al. (2024) [40]	A-PRF	10	8–14 min at 1300 rpm (≈200× *g*)	Advanced PRF prepared at lower speed and longer time; used as membrane or gel.
Aravena et al. (2021) [37] Karagah et al. (2022) [26] Aldommari et al. (2025) [25]	L-PRF	10–20	12 min at 2700–2800 rpm (≈700× *g*)	Leukocyte-rich PRF prepared using standardized Intra-Spin or equivalent systems.
Nagrani et al. (2023) [39]	I-PRF	10	3 min at 700 rpm (≈60× *g*)	Injectable PRF (liquid phase) collected and used for ‘sticky bone.’
Stumbras et al. (2020, 2021) [34,36]	PRGF	Not specified	System-specific centrifugation	PRGF system used to isolate plasma fractions (F1/F2) for membrane formation.
Aldommari et al. (2025) [25]	T-PRF	20	12 min at 2800 rpm (≈700× *g*)	Prepared in titanium tubes; clot compressed into membrane.

PRF—Platelet-rich fibrin; RPM—Rotations per minute; ACD—Acidulated citrate dextrose; A-PRF—Advanced platelet-rich fibrin; PRGF—Plasma rich in growth factors; L-PRF—Leucocyte- and platelet-rich fibrin; I-PRF—Injectable platelet-rich fibrin; T-PRF—Titanium platelet-rich fibrin.

## Data Availability

The original contributions presented in this study are included in the article. Further inquiries can be directed to the corresponding author.

## Supplementary Materials

The following supporting information can be downloaded at: https://www.mdpi.com/article/10.3390/bioengineering12111253/s1. Table S1. Detailed search strings and strategies for searching electronic databases and registries during initial screening. Table S2: Detailed procedural parameters for platelet-rich fibrin preparation protocols in the included studies.

## Abbreviations

The following abbreviations are used in this manuscript:

ARP	Alveolar ridge preservation
PRF	Platelet-rich fibrin
FDBA	Freeze-dried bone allograft
SMD	Standardized mean deviation
CI	Confidence interval
SD	Standard deviation
PICOS	Population, Intervention, Comparator, Outcomes, Study design
PRISMA	Preferred reporting items for systematic reviews and meta-analysis
RCT	Randomized controlled trial
CCT	Controlled clinical trial
RoB	Risk-of-bias
ROBINS	Risk-of-bias for non-randomized studies of interventions
ROBVIS	Risk-of-bias visualization
GRADE	Grading of Recommendations, Assessment, Development and Evaluations
SoF	Summary of findings
CBCT	Cone beam computed tomography
ACD	Acidulated citrate dextrose
RPM	Rotations per minute
RBC	Red blood cell
WBC	White blood cell
PRGF	Plasma rich in growth factors
PPP	Platelet poor plasma

## References

[1] De Risi V., Clementini M., Vittorini G., Mannocci A., De Sanctis M. (2015). Alveolar ridge preservation techniques: A systematic review and meta-analysis of histological and histomorphometrical data. Clin. Oral Implant. Res..

[2] Al-Maawi S., Becker K., Schwarz F., Sader R., Ghanaati S. (2021). Efficacy of platelet-rich fibrin in promoting the healing of extraction sockets: A systematic review. Int. J. Implant Dent..

[3] Alrayyes Y., Aloraini S., Alkhalaf A., Aljasser R. (2022). Soft-Tissue Healing Assessment after Extraction and Socket Preservation Using Platelet-Rich Fibrin (PRF) in Smokers: A Single-Blinded, Randomized, Controlled Clinical Trial. Diagnostics.

[4] Morjaria K.R., Wilson R., Palmer R.M. (2014). Bone healing after tooth extraction with or without an intervention: A systematic review of randomized controlled trials. Clin. Implant. Dent. Relat. Res..

[5] Egierska D., Perszke M., Mazur M., Duś-Ilnicka I. (2023). Platelet-rich plasma and platelet-rich fibrin in oral surgery: A narrative review. Dent. Med. Probl..

[6] Bianchini M.A., Buttendorf A.R., Benfatti C.A., Bez L.V., Ferreira C.F., de Andrade R.F. (2009). The use of freeze-dried bone allograft as an alternative to autogenous bone graft in the atrophic maxilla: A 3-year clinical follow-up. Int. J. Periodontics Restor. Dent..

[7] Alrayyes Y., Al-Jasser R. (2022). Regenerative Potential of Platelet Rich Fibrin (PRF) in Socket Preservation in Comparison with Conventional Treatment Modalities: A Systematic Review and Meta-Analysis. Tissue Eng. Regen. Med..

[8] Alhaj F., Shokry M., Attia N. (2018). The efficiency of using advanced platelet rich fibrin–Autogenous bone graft mixture around immediately placed dental implants in mandibular molar region: (Randomized controlled clinical trial). Egypt. Dent. J..

[9] Azangookhiavi H., Habibzadeh S., Zahmatkesh H., Mellati E., Mosaddad S.A., Dadpour Y. (2024). The effect of platelet-rich fibrin (PRF) versus freeze-dried bone allograft (FDBA) used in alveolar ridge preservation on the peri-implant soft and hard tissues: A randomized clinical trial. BMC Oral Health.

[10] Azangookhiavi H., Ghodsi S., Jalil F., Dadpour Y. (2020). Comparison of the Efficacy of Platelet-Rich Fibrin and Bone Allograft for Alveolar Ridge Preservation after Tooth Extraction: A Clinical Trial. Front. Dent..

[11] Dragonas P., Katsaros T., Avila-Ortiz G., Chambrone L., Schiavo J.H., Palaiologou A. (2019). Effects of leukocyte-platelet-rich fibrin (L-PRF) in different intraoral bone grafting procedures: A systematic review. Int. J. Oral Maxillofac. Surg..

[12] Horváth A., Mardas N., Mezzomo L.A., Needleman I.G., Donos N. (2013). Alveolar ridge preservation. A systematic review. Clin. Oral Investig..

[13] Miron R.J., Fujioka-Kobayashi M., Bishara M., Zhang Y., Hernandez M., Choukroun J. (2017). Platelet-Rich Fibrin and Soft Tissue Wound Healing: A Systematic Review. Tissue Eng. Part B Rev..

[14] Miron R.J., Fujioka-Kobayashi M., Moraschini V., Zhang Y., Gruber R., Wang H.L. (2021). Efficacy of platelet-rich fibrin on bone formation, part 1: Alveolar ridge preservation. Int. J. Oral Implantol..

[15] Alavi S.A., Imanian M., Alkaabi S., Al-Sabri G., Forouzanfar T., Helder M. (2024). A systematic review and meta-analysis on the use of regenerative graft materials for socket preservation in randomized clinical trials. Oral Surg. Oral Med. Oral Pathol. Oral Radiol..

[16] Atieh M.A., Alfardan L., Alsabeeha N.H.M. (2022). Flapped versus flapless alveolar ridge preservation: A systematic review and meta-analysis. Int. J. Oral Maxillofac. Surg..

[17] Canellas J., Soares B.N., Ritto F.G., Vettore M.V., Vidigal Júnior G.M., Fischer R.G., Medeiros P.J.D. (2021). What grafting materials produce greater alveolar ridge preservation after tooth extraction? A systematic review and network meta-analysis. J. Cranio-Maxillo-Facial Surg..

[18] Canellas J.V.d.S., Ritto F.G., Figueredo C.M.d.S., Fischer R.G., de Oliveira G.P., Thole A.A., Medeiros P.J.D. (2020). Histomorphometric evaluation of different grafting materials used for alveolar ridge preservation: A systematic review and network meta-analysis. Int. J. Oral Maxillofac. Surg..

[19] Liu M., Liu Y., Luo F. (2023). The role and mechanism of platelet-rich fibrin in alveolar bone regeneration. Biomed. Pharmacother..

[20] Page M.J., McKenzie J.E., Bossuyt P.M., Boutron I., Hoffmann T.C., Mulrow C.D., Shamseer L., Tetzlaff J.M., Akl E.A., Brennan S.E. (2021). The PRISMA 2020 statement: An updated guideline for reporting systematic reviews. BMJ (Clin. Res. Ed.).

[21] Sterne J.A., Hernán M.A., Reeves B.C., Savović J., Berkman N.D., Viswanathan M., Henry D., Altman D.G., Ansari M.T., Boutron I. (2016). ROBINS-I: A tool for assessing risk of bias in non-randomised studies of interventions. BMJ (Clin. Res. Ed.).

[22] Sterne J.A.C., Savović J., Page M.J., Elbers R.G., Blencowe N.S., Boutron I., Cates C.J., Cheng H.Y., Corbett M.S., Eldridge S.M. (2019). RoB 2: A revised tool for assessing risk of bias in randomised trials. BMJ (Clin. Res. Ed.).

[23] McGuinness L.A., Higgins J.P.T. (2021). Risk-of-bias VISualization (robvis): An R package and Shiny web app for visualizing risk-of-bias assessments. Res. Synth. Methods.

[24] Guyatt G.H., Oxman A.D., Vist G.E., Kunz R., Falck-Ytter Y., Alonso-Coello P., Schünemann H.J. (2008). GRADE: An emerging consensus on rating quality of evidence and strength of recommendations. BMJ (Clin. Res. Ed.).

[25] Aldommari E.A., Omair A., Qasem T. (2025). Titanium-prepared platelet-rich fibrin enhances alveolar ridge preservation: A randomized controlled clinical and radiographic study. Sci. Rep..

[26] Karagah A., Tabrizi R., Mohammadhosseinzade P., Mirzadeh M., Tofangchiha M., Lajolo C., Patini R. (2022). Effect of Sinus Floor Augmentation with Platelet-Rich Fibrin Versus Allogeneic Bone Graft on Stability of One-Stage Dental Implants: A Split-Mouth Randomized Clinical Trial. Int. J. Environ. Res. Public Health.

[27] Tajima N., Ohba S., Sawase T., Asahina I. (2013). Evaluation of sinus floor augmentation with simultaneous implant placement using platelet-rich fibrin as sole grafting material. Int. J. Oral Maxillofac. Implant..

[28] Girish Rao S., Bhat P., Nagesh K.S., Rao G.H., Mirle B., Kharbhari L., Gangaprasad B. (2013). Bone regeneration in extraction sockets with autologous platelet rich fibrin gel. J. Maxillofac. Oral Surg..

[29] Thakkar D.J., Deshpande N.C., Dave D.H., Narayankar S.D. (2016). A comparative evaluation of extraction socket preservation with demineralized freeze-dried bone allograft alone and along with platelet-rich fibrin: A clinical and radiographic study. Contemp. Clin. Dent..

[30] Alzahrani A.A., Murriky A., Shafik S. (2017). Influence of platelet rich fibrin on post-extraction socket healing: A clinical and radiographic study. Saudi Dent. J..

[31] Clark D., Rajendran Y., Paydar S., Ho S., Cox D., Ryder M., Dollard J., Kao R.T. (2018). Advanced platelet-rich fibrin and freeze-dried bone allograft for ridge preservation: A randomized controlled clinical trial. J. Periodontol..

[32] Zhang Y., Ruan Z., Shen M., Tan L., Huang W., Wang L., Huang Y. (2018). Clinical effect of platelet-rich fibrin on the preservation of the alveolar ridge following tooth extraction. Exp. Ther. Med..

[33] Taha M. (2019). Short term dimensional bony changes following teeth extraction in the esthetic zone and the use of PRF as a sole grafting material: Randomized controlled trial. Egypt. J. Oral Maxillofac. Surg..

[34] Stumbras A., Januzis G., Gervickas A., Kubilius R., Juodzbalys G. (2020). Randomized and Controlled Clinical Trial of Bone Healing After Alveolar Ridge Preservation Using Xenografts and Allografts Versus Plasma Rich in Growth Factors. J. Oral Implantol..

[35] Ivanova V., Chenchev I., Zlatev S., Mijiritsky E. (2021). Comparison Study of the Histomorphometric Results after Socket Preservation with PRF and Allograft Used for Socket Preservation-Randomized Controlled Trials. Int. J. Environ. Res. Public Health.

[36] Stumbras A., Galindo-Moreno P., Januzis G., Juodzbalys G. (2021). Three-dimensional analysis of dimensional changes after alveolar ridge preservation with bone substitutes or plasma rich in growth factors: Randomized and controlled clinical trial. Clin. Implant Dent. Relat. Res..

[37] Aravena P.C., Sandoval S.P., Pizarro F.E., Simpson M.I., Castro-Adams N., Serandour G., Rosas C. (2021). Leukocyte and Platelet-Rich Fibrin Have Same Effect as Blood Clot in the 3-Dimensional Alveolar Ridge Preservation. A Split-Mouth Randomized Clinical Trial. J. Oral Maxillofac. Surg..

[38] Niedzielska I., Ciapiński D., Bąk M., Niedzielski D. (2022). The Assessment of the Usefulness of Platelet-Rich Fibrin in the Healing Process Bone Resorption. Coatings.

[39] Nagrani T., Kumar S., Haq M.A., Dhanasekaran S., Gajjar S., Patel C., Sinha S., Haque M. (2023). Use of Injectable Platelet-Rich Fibrin Accompanied by Bone Graft in Socket Endurance: A Radiographic and Histological Study. Cureus.

[40] Khaddour A.S., Ghiță R.E., Ionescu M., Rîcă R.G., Mercuț V., Manolea H.O., Camen A., Drăghici E.C., Radu A., Popescu S.M. (2024). Healing of Extraction Sites after Alveolar Ridge Preservation Using Advanced Platelet-Rich Fibrin: A Retrospective Study. Bioengineering.

[41] Molina-Barahona M., Castillo J., Freire-Meza E., Vásquez-Palacios A.C., Morales-Navarro D., Avecillas-Rodas R. (2025). Radiographic Evaluation in Alveolar Preservation Using Platelet-Rich Fibrin: A Randomized Controlled Trial. Dent. J..

[42] Khan J., Bandi S., Gangineni S., Kummari S., Pradeep D.G., Hinduja T. (2024). Evaluation of Alveolar Ridge Dimensions by Socket Preservation Therapy Using a Bone Graft and Platelet-Rich Fibrin: A Randomized Controlled Trial. Cureus.

[43] Wardani A., Tran B., Duterre M., Larabi I., Waskiewicz K., Louryan S., Evrard L. (2023). Healing of particulate allografts mixed with platelet concentrates in ridge preservation and sinus lift: A prospective histomorphometric study. Morphologie.

[44] Arumugam P., Baburaj M.D., Yadalam P.K., Ardila C.M. (2025). A comparative study of platelet-rich fibrin plugs versus biphasic calcium phosphate in treating infrabony defects in patients with periodontitis: Insights from a randomized controlled trial. J. Clin. Exp. Dent..

[45] Borg T.D., Mealey B.L. (2015). Histologic healing following tooth extraction with ridge preservation using mineralized versus combined mineralized-demineralized freeze-dried bone allograft: A randomized controlled clinical trial. J. Periodontol..

[46] Pan J., Xu Q., Hou J., Wu Y., Liu Y., Li R., Pan Y., Zhang D. (2019). Effect of platelet-rich fibrin on alveolar ridge preservation: A systematic review. J. Am. Dent. Assoc..

[47] Garg M., Srivastava V., Chauhan R., Pramanik S., Khanna R. (2023). Application of platelet-rich fibrin and freeze-dried bone allograft following apicoectomy: A comparative assessment of radiographic healing. Indian J. Dent. Res..

[48] Madi M., Almindil I., Alrassasi M., Alramadan D., Zakaria O., Alagl A.S. (2023). Cone-Beam Computed Tomography and Histological Findings for Socket Preservation Techniques Using Different Grafting Materials: A Systematic Review. J. Funct. Biomater..

[49] Philip M.R., AlOtaibi S., AlEid B. (2022). The success rates of various surgical techniques for socket preservation in the aesthetic zone: A systematic review and meta-analysis. J. Oral Maxillofac. Surg. Med. Pathol..

[50] Razi M.A., Mahajan A., Zarrin R., Roy S., Singh M.K., Kumari S. (2024). Impact of Platelet-Rich Fibrin (PRF) Versus Freeze-Dried Bone Allograft (FDBA) on Peri-Implant Soft and Hard Tissue in Alveolar Ridge Preservation. J. Pharm. Bioallied Sci..

[51] Dhamija R., Shetty V., Vineeth K., Nagaraju R., Rao R.S. (2020). Socket preservation with demineralized freeze-dried bone allograft and platelet-rich fibrin for implant site development: A randomized controlled trial. J. Indian Prosthodont. Soc..

[52] Beldhi M., Penmetsa G.S., Gottumukkala S., Ramesh K.S.V., Kumar P.M., Manchala B. (2024). Evaluation and comparison of autologous particulate dentin with demineralized freeze dried bone allograft in ridge preservation procedures—A prospective clinical study. Clin. Oral Investig..

[53] Liu Y., Li X., Jiang C., Guo H., Luo G., Huang Y., Yuan C. (2022). Clinical applications of concentrated growth factors membrane for sealing the socket in alveolar ridge preservation: A randomized controlled trial. Int. J. Implant Dent..

[54] Elboraey M.O., Alqutaibi A.Y., Aboalrejal A.N., Borzangy S., Zafar M.S., Al-Gabri R., Alghauli M.A., Ramalingam S. (2025). Regenerative approaches in alveolar bone augmentation for dental implant placement: Techniques, biomaterials, and clinical decision-making: A comprehensive review. J. Dent..

[55] Baniasadi B., Evrard L. (2017). Alveolar Ridge Preservation After Tooth Extraction with DFDBA and Platelet Concentrates: A Radiographic Retrospective Study. Open Dent. J..

[56] Al-Badran A., Bierbaum S., Wolf-Brandstetter C. (2023). Does the Choice of Preparation Protocol for Platelet-Rich Fibrin Have Consequences for Healing and Alveolar Ridge Preservation After Tooth Extraction? A Meta-Analysis. J. Oral Maxillofac. Surg..

[57] Elsheikh H.A.-E., Abdelsameaa S.E., Elbahnasi A.A., Abdel-Rahman F.H. (2023). Comparison between platelet rich fibrin as space filling material versus xenograft and alloplastic bone grafting materials in immediate implant placement: A randomized clinical trial. BMC Oral Health.

[58] Agarwal A., Gupta N.D., Jain A. (2016). Platelet rich fibrin combined with decalcified freeze-dried bone allograft for the treatment of human intrabony periodontal defects: A randomized split mouth clinical trail. Acta Odontol. Scand..

[59] Sherif M.A., Anter E., Graetz C., El-Sayed K.F. (2025). Injectable platelet-rich fibrin with vitamin C as an adjunct to non-surgical periodontal therapy in the treatment of stage-II periodontitis: A randomized controlled clinical trial. BMC Oral Health.

