# Supplementary material for: Comparative Efficacy of Platelet-Rich Fibrin, Freeze-Dried Bone Allograft, or Spontaneous Healing for Alveolar Ridge Preservation: Systematic Review and Meta-Analysis
Source: Bioengineering (Basel). 2025 Nov 16;12(11):1253. doi: 10.3390/bioengineering12111253 (PMC12650340; doi:10.3390/bioengineering12111253)
Supplement: Supplementary file 1 [file bioengineering-12-01253-s001.zip › bioengineering-3929186-supplementary/Supplemental Table - S1.pdf]

Table S1. Detailed search strings and strategies for searching electronic databases and registries during initial screening.

Electronic Database / Registry	Search String (with Boolean operators)	Date of Search	Limits/Filters Applied	Search Results
PubMed / MEDLINE	("platelet-rich fibrin"[Mesh] OR "platelet rich fibrin" OR PRF) AND ("freeze-dried bone allograft" OR "FDBA" OR "bone graft" OR "bone allograft" OR "demineralized freeze-dried bone allograft") AND ("alveolar ridge preservation" OR "ridge preservation" OR "socket preservation" OR "tooth extraction socket" OR "post-extraction ridge") AND ("randomized controlled trial"[Publication Type] OR "controlled clinical trial" OR "comparative study")	30 June 2025	▪ Human studies<br>▪ English language	14
Cochrane CENTRAL	("platelet-rich fibrin" OR PRF) AND ("freeze-dried bone allograft" OR "FDBA" OR "bone allograft") AND ("alveolar ridge preservation" OR "socket preservation" OR "tooth extraction socket")	30 June 2025	▪ Clinical trials<br>▪ English language	20
Web of Science (All databases)	("platelet-rich fibrin" OR PRF) AND ("freeze-dried bone allograft" OR "FDBA" OR "bone allograft") AND ("alveolar ridge preservation" OR "socket preservation" OR "tooth extraction socket")	30 June 2025	▪ Human studies<br>▪ English language	106
ScienceDirect (Elsevier)	TITLE-ABS-KEY ("platelet-rich fibrin" OR PRF) AND TITLE-ABS-KEY ("freeze-dried bone allograft" OR FDBA OR "bone allograft") AND TITLE-ABS-KEY ("alveolar ridge preservation" OR "socket preservation" OR "extraction socket")	30 June 2025	▪ Human studies<br>▪ English language	51
Google Scholar	"platelet-rich fibrin" OR PRF AND "freeze-dried bone allograft" OR FDBA AND "alveolar ridge preservation" OR "socket preservation" OR "tooth extraction socket"	30 June 2025	▪ First 400 results<br>▪ Human studies<br>▪ English language	400

Sequence of study search strategy and selection:

- According to the database and its indexing, the search strings syntaxes were adapted and Boolean operators (AND, OR) and field tags (e.g., [Mesh], TS=, TITLE-ABS-KEY) were used appropriately.
- Studies identified through searching were imported to a reference management software (EndNote) for identification of duplicates, and title and abstract screening.
- Study search and selection was conducted by two independently calibrated reviewers (A.S.A. and A.M.B.; with a Cohen's kappa score of 0.87). Discrepancies, if any, were resolved by consensus or through consultation with a third reviewer (S.R.).
