# Supplementary material for: Comparative Efficacy of Platelet-Rich Fibrin, Freeze-Dried Bone Allograft, or Spontaneous Healing for Alveolar Ridge Preservation: Systematic Review and Meta-Analysis
Source: Bioengineering (Basel). 2025 Nov 16;12(11):1253. doi: 10.3390/bioengineering12111253 (PMC12650340; doi:10.3390/bioengineering12111253)
Supplement: Supplementary file 1 [file bioengineering-12-01253-s001.zip › bioengineering-3929186-supplementary/Supplemental Table - S2.pdf]

Table S2: Detailed procedural parameters for platelet-rich fibrin preparation protocols in the included studies.

Author (year)	PRF Type	Blood Volume (per preparation)	Anticoagulant	Tube Type	Centrifugation Parameters	Brief Description of Synthesis Technique
Tajima et al. (2013)	PRF clot	9 mL	No	9 mL sterile glass tubes	Specialized centrifuge using multi-stage program: 30 sec acceleration, 2 min at 2,700 rpm, 4 min at 2,400 rpm, 4 min at 2,700 rpm, 3 min at 3,000 rpm, 30 sec deceleration.	**Blood drawn → centrifuged immediately**<br>• Fibrin clot forms between serum and RBC layer<br>• Clot removed and separated from RBC base
Girish Rao et al. (2013)	PRF gel	4.5 mL	Yes (0.5 mL ACD)	Sterile glass tube containing 0.5 ml ACD	20 min at 360–400 rpm	**Blood mixed with ACD → centrifuged**<br>• Top PPP discarded<br>• Middle PRP collected → mixed with calcium gluconate<br>• Left 10 min → PRF gel formed
Thakkar et al. (2016)	PRF clot	10 mL	No	10 ml syringe	10 min at 3000 rpm	**Blood collected → centrifuged**<br>• Fibrin clot obtained<br>• Clot transferred into socket
Alzahrani et al. (2017)	PRF membrane	20 mL	No	Not specified	10 min at 3000 rpm (approx. 400 x g)	**Blood drawn → centrifuged immediately**<br>• Fibrin clot removed, RBCs scraped off<br>• Clot compressed in PRF box → fibrin membrane
Clark et al. (2018)	A-PRF clot	10 mL	No	Sterile glass vacuum tube	8 min at 1,300 rpm (approx. 200 x g)	**Venous blood drawn → centrifuged at low speed**<br>• A-PRF clot separated from 3 distinct layers
Zhang et al. (2018)	PRF membrane	9 mL	No	Glass test tubes	10 min at approx. 400 x g	**Blood centrifuged**<br>• Middle PRF gel layer removed<br>• RBC layer discarded<br>• Gel pressed lightly in PRF box → elastic PRF membrane
Taha (2019)	PRF	Not specified	No	10 mL sterile glass tubes	10 min at 3000 rpm	**Blood drawn → centrifuged immediately**<br>• PRF separated from RBCs<br>• PRF transferred into extraction site

Azangookhiavi et al. (2020, 2024)	PRF membrane/ clot	10 mL	No	Not specified	12 min at 2700 rpm	**Blood obtained → centrifuged**<br>• 1–3 PRF clots placed in socket<br>• Covered with PRF membrane
Stumbras et al. (2020, 2021)	PRGF	Not specified	No	PRGF System Centrífuge-IV tubes	Centrifuged in PRGF System Centrífuge-IV	**Blood centrifuged → 3 fractions separated (RBCs, WBCs, plasma)**<br>• F1 (top plasma layer) → fibrin membrane<br>• F2 (above leukocyte layer) → platelet-rich fraction
Ivanova et al. (2021)	PRF membrane/ clot	Not specified	No	Not specified	12 min at 3000 rpm	**Blood centrifuged**<br>• Fibrin clot obtained (stable, elastic, flexible)<br>• Used as graft material or compressed into membrane
Aravena et al. (2021)	L-PRF	10 mL	No	10 mL sterile glass tubes	12 min at 2700 rpm (approx. 700 x g)	**Blood drawn → centrifuged immediately**<br>• L-PRF prepared using previously established protocol
Alrayyes et al. (2022)	A-PRF membrane	10 mL	No	Sterile glass vacuum tube	14 min at 1300 rpm	**A-PRF protocol**<br>• Blood centrifuged → fibrin clot compressed in PRF box → membrane<br>**S-PRF protocol**<br>• Liquid layer collected → mixed with FDBA → sticky bone
Karagah et al. (2022)	L-PRF membrane	20 mL	No	Intra-spin centrifuge tubes of Intra-Lock machine	12 min at 2800 rpm	**Blood centrifuged immediately**<br>• L-PRF clot separated from RBCs<br>• Placed in Xpression tray 5 min → PRF membrane
Niedzielska et al. (2022)	PRF clot	18 mL (incisors/premolars); 27 mL (molars)	No	9 mL sterile glass tubes	12 min at 2700 rpm	**Blood collected → centrifuged immediately**<br>• PRF clot separated from RBCs<br>• Inserted into alveolus
Nagrani et al. (2023)	I-PRF	10 mL	No	Plastic tubes	3 min at 700 rpm (approx. 60 x g)	**Blood centrifuged at low speed**<br>• Top liquid layer collected → I-PRF<br>• I-PRF mixed with graft (DFDBA/FDBA) → sticky bone

Khaddour et al. (2024)	A-PRF	10 mL	No	Sterile glass vacuum tube	14 min at 1300 rpm	**Blood collected → centrifuged immediately**<br>• A-PRF gel (buffy coat) isolated<br>• Half compressed into membrane, half used as gel
Aldommari et al. (2025)	T-PRF	20 mL	No	Sterile Grade IV titanium tubes	12 min at 2800 rpm (approx. 708 x g)	**Blood collected in titanium tubes → centrifuged**<br>• Clot removed, RBCs scraped off<br>• Clot pressed → fibrin membrane<br>• 1–2 membranes placed into sockets
	L-PRF	18 mL	No	Sterile glass vacuum tube	12 min at 2700 rpm (approx. 700 x g)	**Blood collected in glass tubes → centrifuged**<br>• Clot removed, RBCs scraped off<br>• Clot pressed → fibrin membrane<br>• 1–2 membranes placed into sockets
Molina-Barahona et al. (2025)	PRF membrane/ clot	10 mL	No	Sterile glass vacuum tube	12 min at 2700 rpm	**Blood collected → centrifuged immediately**<br>• PRF clot extracted (between RBCs and plasma)<br>• One clot filled socket, second flattened as membrane

PRF – Platelet-rich fibrin; RPM – Rotations per minute; RBC – Red blood cell; ACD – Acidulated citrate dextrose; PPP – Platelet poor plasma; A-PRF – Advanced platelet-rich fibrin; PRGF – Plasma rich in growth factors; WBC – White blood cell; L-PRF – Leucocyte and platelet-rich fibrin; S-PRF – Sticky bone- platelet-rich fibrin; FDBA – Freeze-dried bone allograft; I-PRF – Injectable platelet-rich fibrin; T-PRF – Titanium platelet-rich fibrin
